# Oxygen-enhanced MRI can accurately identify, quantify and map tumour hypoxia in preclinical models

**DOI:** 10.1186/1470-7330-15-S1-P9

**Published:** 2015-10-02

**Authors:** JPB O'Connor, JKR Boult, Y Jamin, M Babur, KG Finegan, KJ Williams, AR Reynolds, RA Little, A Jackson, GJM Parker, JC Waterton, SP Robinson

**Affiliations:** 1 University of Manchester, Oxford Road, Manchester, M13 9PL, UK

## Aim

There is need for non-invasive methods to identify, quantify and map tumour hypoxia. In this study we used an emerging technology – R_1_ oxygen enhanced MRI (OE-MRI) – to distinguish those tumour sub-regions that respond to hyperoxic gas challenge from refractory sub-regions. We hypothesised that the proportion of refractory tumour tissue (Oxy-R) would be a robust biomarker of tumour hypoxia across multiple models with different vascular and hypoxic phenotypes.

Methods: OE-MRI signal precision, stability and relationship to tissue pO_2_ were evaluated in well vascularised renal cancer 786-O xenografts. Dynamic sensitivity of proportional Oxy-R to acute changes in hypoxia was evaluated using hydralazine challenge. Relationship of proportional Oxy-R to tissue immunohistochemistry and gadolinium DCE-MRI were explored in parental and drug-resistant 786-O models and in SW620 xenografts.

## Results

Phantom and *in vivo* experiments demonstrated the accuracy, precision and stability of R_1_ measurement. The proportion of tumour Oxy-R increased significantly following hydralazine challenge (p=0.045) relative to control. The proportion of tumour with perfused Oxy-R voxels was correlated to chronic hypoxia in well perfused 786-O-R xenografts(rho 0.810, p=0.028) and in relatively necrotic SW620 xenografts (rho 0.929, p=0.002).

## Conclusion

The proportion of tumour perfused Oxy-R is a robust biomarker of tumour hypoxia. Voxel-wise analysis of dual oxygen and gadolinium challenge has potential to quantify and map tumour hypoxia as prognostic, predictive and pharmacodynamic biomarkers that could facilitate personalised healthcare.

